# Joining the meta-research movement: A bibliometric case study of the journal *Perspectives on Medical Education*

**DOI:** 10.1007/s40037-022-00717-9

**Published:** 2022-06-21

**Authors:** Lauren A. Maggio, Stefanie Haustein, Joseph A. Costello, Erik W. Driessen, Anthony R. Artino Jr

**Affiliations:** 1 Uniformed Services University of the Health Sciences, Bethesda, MD USA; 2 School of Information Studies (ÉSIS) and Scholarly Communications Lab, University of Ottawa, Ottawa, ON Canada; 3 Henry M. Jackson Foundation, Bethesda, MD USA; 4 Maastricht University, Maastricht, The Netherlands; 5 The George Washington University School of Medicine and Health Sciences, Washington, DC, USA

**Keywords:** Scholarly communication, Bibliometrics, Journals, Meta-research

## Abstract

**Purpose:**

To conduct a bibliometric case study of the journal *Perspectives on Medical Education* (PME) to provide insights into the journal’s inner workings and to “take stock” of where PME is today, where it has been, and where it might go.

**Methods:**

Data, including bibliographic metadata, reviewer and author details, and downloads, were collected for manuscripts submitted to and published in PME from the journal’s Editorial Manager and Web of Science. Gender of authors and reviewers was predicted using Genderize.io. To visualize and analyze collaboration patterns, citation relationships and term co-occurrence social network analyses (SNA) were conducted. VOSviewer was used to visualize the social network maps.

**Results:**

Between 2012–2019 PME received, on average, 260 manuscripts annually (range = 73–402). Submissions were received from authors in 81 countries with the majority in the United States (US), United Kingdom, and the Netherlands. PME published 518 manuscripts with authors based in 31 countries, the majority being in the Netherlands, US, and Canada. PME articles were downloaded 717,613 times (mean per document: 1388). In total 1201 (55% women) unique peer reviewers were invited and 649 (57% women) completed reviews; 1227 (49% women) unique authors published in PME. SNA revealed that PME authors were quite collaborative, with most authoring articles with others and only a minority (*n* = 57) acting as single authors.

**Discussion:**

This case study provides a glimpse into PME and offers evidence for PME’s next steps. In the future, PME is committed to growing the journal thoughtfully; diversifying and educating editorial teams, authors, and reviewers, and liberating and sharing journal data.

## Introduction

In 2005, Ioannidis described the scientific community’s increasing concern that most research findings are false due to poor research practices [1]. This article and the resulting media coverage helped marshal scientists to examine how research is performed, communicated, verified, and rewarded, and it spurred organizations to fund these efforts [2, 3]. In other words, this single article helped mobilize the study of research itself, and the meta-research movement (aka, the metascience movement) began in earnest [3]. This movement has exposed a multitude of issues, including irresponsible research practices [4–6], bias in peer review [7], lengthy publication timelines [8, 9], and restricted access to disseminated research [10]. It also has encouraged researchers, journals, and funding agencies to begin seeking transdisciplinary approaches to mitigating some of these challenges.

Many organizations, including academic institutions, journals, and publishers, have taken the findings of meta-research seriously and have tried to improve their processes and incentives. For example, the *British Medical Journal* (BMJ) conducts meta-research across its suite of journals and offers a doctoral program on the responsible conduct of research. PLOS, which strives to empower researchers to accelerate scientific progress by leading a transformation in science communication, takes a similar approach [11]. Moreover, PLOS readily makes available its articles and related metadata to be mined, shared, and reused, which facilitates meta-research and increases the transparency of its content and processes [12]. Similar to these journals, *Perspectives on Medical Education* (*PME*) also strives to take meta-research seriously and apply findings from its articles to improve journal processes and content.

One approach to meta-research is bibliometrics, which is the use of statistical methods to analyze publications. The field of bibliometrics is well-established, dating back to 1934 [13] and popularized in the 1960s with the advent of what is now Web of Science [14]. When used carefully, in appropriate contexts and complementary to peer evaluation, bibliometrics can be a powerful way to understand publication and citation behavior of entire countries, research fields or journals using large-scale statistical analyses [15].

To open the special issue on meta-research in medical education, this article uses bibliometrics to undertake a case study of PME itself. In it, we hope to provide readers, authors, reviewers, and editors with transparency about the journal’s inner workings and offer insights into the context of the scholarly conversation taking place in PME. Our aim is to make this analysis useful to different stakeholders with, for example, their interpretation of scholarly discussions or editorial decisions. Additionally, we wrote this article as a way of “taking stock” of where we are today, where we have been in the past, and where we might go over the next decade, with the hopes of using an evidence-based approach to guide the journal’s future. Lastly, as we present our results and interpretations in this paper, we pose a series of questions and invite the health professions education (HPE) community to begin a dialogue on these important meta-research issues, and we encourage members of the HPE community to think about how the journal and the field could move forward.

## Methods

We conducted a bibliometric case study of PME using bibliographic metadata, citations, downloads, and statistics about the submission and review process. As journals are complex entities that generate a wealth of data, for feasibility we choose to focus on several key aspects that we feel are relevant for PME’s readers, authors, reviewers, and editors. In particular, we provide an overview of the manuscripts submitted to and published in the journal, including descriptions of publication types, author characteristics, related author keywords, and overall acceptance rates. We also examine peer-review practices, such as publication timelines and reviewer characteristics and, for published articles, we describe article usage and citation data. Finally, using social network analysis, we explore the relationships between authors, institutions, and topics.

### About PME

PME is the official journal of the Netherlands Association of Medical Education (NVMO). The journal’s mission is to support and enrich collaborative scholarship between education researchers and clinical educators, and to advance new knowledge regarding clinical education practices by publishing a variety of publication types including original research. The NVMO’s sponsorship enables PME to be a diamond Open Access journal, which levies no author charges and makes all articles freely available immediately under a Creative Commons attribution license. This is the least restrictive Creative Commons license enabling reuse while still providing credit to the authors. PME is published by Bohn Stafleu van Loghum, part of Springer Nature, and indexed in over 20 databases, including MEDLINE, Web of Science (WoS), and Embase. In 2020, PME was recognized with the Directory of Open Access Journals Seal of Approval for Open Access Journals and was also awarded a journal impact factor by WoS. PME is a single-blind, peer-reviewed journal. Manuscripts are handled by an associate editor and reviewed by at least two reviewers. The editor-in-chief renders all final decisions.

### Data collection

Data for this case study were collected for manuscripts submitted and published between 2012–2019. We focused on this time period because after 2012 PME transitioned from a Dutch to an English language journal and established an international editorial board. We primarily extracted data from PME’s version of Editorial Manager with the exception of usage data, which were provided by Springer, and basic citation data, which were obtained via WoS.

Author names, institutions, and countries were cleaned to combine various spellings and variants of the same entity into one (e.g., UCSF was combined with University of California, San Francisco). This was done using the VOSviewer thesaurus function. For author names we changed 58 spellings of author names for 53 authors to merge them with alternative spelling variants. The majority of changes affected first name initials only (e.g., “driessen, e” to “driessen, ew”). Author name disambiguation reduced the total number of distinct authors from 1285 to 1227. We also cleaned institution names, reducing the total number of institutions from 423 to 333. We found 955 keywords and, for ease of analysis, selected only those that appeared at least three times in our dataset, which reduced the keywords most frequently found to 101.

To predict the gender of authors and peer reviewers, we used the tool Genderize.io [16], which calculates a probability score for whether each first name is more likely to refer to a woman or man. If the tool predicted with 70% confidence that a given name was a given gender, we accepted the designation. For those names with less certainty or unknown gender (*n* = 62), we searched the individuals’ online presence. We recognize that gender is a complex social construct that is best described by an individual and that the binary nature of these results is not ideal. However, because the field of bibliometrics currently lacks better alternatives, we decided to use this approach, which has been employed to predict gender in several recent studies with similar aims [17–19].

### Analysis

Descriptive statistics were calculated using GoogleSheets and Excel [20, 21]. To visualize and analyze collaboration patterns, citation relationships, and term co-occurrence, we conducted social network analyses. We used VOSviewer [22] to extract network data from the bibliographic metadata downloaded from WoS and to visualize the networks. Layout and cluster resolution settings vary depending on the size and structure of the particular network in order to optimize the network graphs [23].

Our author team includes researchers with expertise in HPE and information science. Three of the authors have official connections to PME. ED is the Editor-in-Chief, LM is the Deputy Editor-in-Chief, and AA is an Associate Editor of PME. SH is a guest editor for PME’s special issue on meta-research. We recognize that our relationship with the journal gives us information access that is traditionally not publicly available (e.g. usage data); this is a strength of this case study. However, we also realize that this could be construed as a conflict of interest. Therefore, for transparency, we deposited the study data obtained from Editorial Manager in an open-access database to allow for independent review and replication of our findings [23]. Moreover, in the results and discussion sections, we propose some interpretation of the data based on our own experience as editors and researchers in the field. However, we recognize that these interpretations are subject to our own biases and that other researchers may draw different conclusions. Thus, our aim is not to provide a definitive interpretation of these network maps, but rather to invite the HPE community to draw their own conclusions and to engage in a broader discussion. To that end, we have embedded questions to the community for each of the visualizations to spark thought and encourage dialogue. These questions are labeled: For Discussion.

## Results and discussion

Although we were fortunate to have access to a great deal of data, we are mindful that journal pages are a precious and finite resource. Therefore, we provide here only a summary of our findings and have deposited supplementary tables and figures as freely accessible resources on Zenodo [23], and reference these resources throughout.

**Fig. 1:**
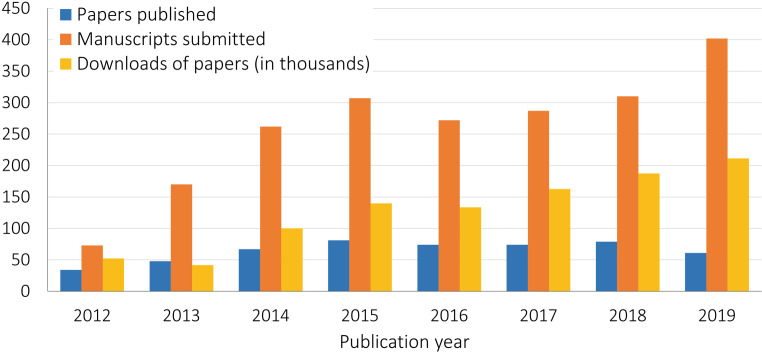
Number of annual submissions, publications and downloads (2012–2019)

PME received an average of 260 manuscripts per year (median = 280; range = 73–402; SD = 99). From 2012–2019 there was a 451% increase in submissions (Fig. 1). The overall annual acceptance rate of manuscripts has trended down over time: in 2012, the acceptance rate was 46.6%; in 2019, the acceptance rate was just 15.2%. Over this time, the total number of accepted manuscripts has stayed relatively constant.

PME received manuscripts from authors based in 81 countries. Corresponding authors for submitted manuscripts were primarily based in the United States (US) (*n* = 514, 24.7%), United Kingdom (UK) (*n* = 312; 15.0%), and the Netherlands (*n* = 276; 13.3%). Manuscripts submitted by authors based in the Netherlands had the highest acceptance rate (25.5%) followed by authors in the US (22.6%) and Canada (22.3%). See the dataset on Zenodo for counts of manuscripts accepted and submitted across all countries [23].

Between 2012–2019, PME published 518 articles based on 2082 submissions, for an overall acceptance rate of 24.9%. On average, PME published 65 articles per year (median = 71). Each issue contained, on average, 11 articles (median = 11; SD = 2.5) with six issues published annually during most years. A seventh supplementary special issue published in 2018 focused on researchers’ surprises and failures [24].

The majority of publications were research articles (62.9%), followed by editorial material (24.5%), reviews (5.8%), and letters (5.8%). Two book reviews and two corrections were also published. While review articles became more frequent in 2017, fewer letters were published in 2016 and beyond.

PME articles were downloaded 717,613 times (per document downloads: mean = 1388.0; median = 1010; SD = 1468.2). Despite being recently published in 2019, an article by Neubauer et al. [34] that provides practical instruction on using phenomenology, a qualitative method, was the most downloaded article at the time of this analysis, in terms of absolute numbers (17,313 downloads) and when normalized by time relative to issues. The next most downloaded article was a paper from the journal’s Writer’s Craft series (12,677 downloads), which provides authors helpful tips for improving their writing [35]. See Tab. 1 for the top 5 most downloaded articles.

**Table 1:** Top 5 articles with highest number of downloads

First authors (year)	Title	Downloads
Neubauer (2019) [34]	How phenomenology can help us learn from the experiences of others	17,313
Varpio (2018) [35]	Using rhetorical appeals to credibility, logic, and emotions to increase your persuasiveness	12,677
Kamphuis (2014) [39]	Augmented reality in medical education?	9928
Lefroy (2015) [37]	Guidelines: the do’s, don’ts and don’t knows of feedback for clinical education	9091
Kogan (2017) [40]	Guidelines: The do’s, don’ts and don’t knows of direct observation of clinical skills in medical education	8470

The most frequently cited PME articles in terms of absolute number of citations were Artino [36], Lefroy et al. [37], and Peters et al. [38], which all received 80 citations or more. These three articles are all forms of literature reviews, which are noted for having high citation rates when compared with original research articles [25]. It is worth noting that all three of these articles were published 5 years ago or longer, and so their relatively high citation rates make intuitive sense because citations accumulate over time. In light of this time dependence, a common bibliometric method is to normalize citations by publication year and to compare relative citation rates. To do this, each article’s absolute citations are divided by the average number of citations of all papers published in the same year (and often in the same field). Such a normalized citation rate is generally considered a fairer approach to comparing the citation impact of articles published in different years. Using the normalized citation rate, the most frequently cited PME article is the article on phenomenology [34]. Tab. 2 lists the top 5 articles that have been cited most frequently; it also includes the citations, normalized for publication age.

**Table 2:** Top 5 articles with highest number of normalized citations

First authors (year)	Title	Citations (absolute number)	Citations (normalized per year)
Neubauer (2019) [34]	How phenomenology can help us learn from the experiences of others	25	14.2
Lefroy (2015) [37]	Guidelines: the do’s, don’ts and don’t knows of feedback for clinical education	83	11.4
Leppink (2015) [41]	The evolution of cognitive load theory and its application to medical education	72	9.9
Peters (2014) [38]	Bedside teaching in medical education: a literature review	83	8.7
Artino (2012) [36]	Academic self-efficacy: from educational theory to instructional practice	85	7.4

### Reviewers

Between 2012–2019, 1201 unique peer scholars were invited to review manuscripts. Of those, 649 (54.0%) completed reviews, with the slight majority reviewing only a single manuscript (*n* = 369). On average, reviewers reviewed 2.78 manuscripts over the study period (range 1–35).

Reviewers were based in 49 countries with those in the United States (US) invited most frequently (*n* = 325; 27.1%) followed by the Netherlands (*n* = 217; 18.1%) and Canada (*n* = 200; 16.7%). Women were invited to review more often than men (women *n* = 655; 54.5%) and completed more reviews (*n* = 369, 56.7%).

On average, reviewers took 16.1 days from accepting a review assignment to submitting their final review (range = 0–82). Women took on average 16.7 days to submit a review (range = 0–82; median = 17; SD = 10.4) and men 15.4 days (range 0–65; median = 16; SD = 11). Values of 0 indicate that the reviewers returned their reviews on the same day that they received their assignments.

### Authors

A total of 1227 unique authors published in PME between 2012–2019. Overall, 49% of authors were predicted to be women and 46% men. We were unable to determine the gender of 5% of names. Men and women were similarly productive (1.4 vs. 1.3 documents per author, respectively) and collaborative (5.0 vs. 4.7 co-authors per author, respectively), which aligns with related findings suggesting relative gender parity in HPE publications in recent years [26, 27]. The most productive authors were Lingard with 20 and Leppink with 16 publications (see Tab. 3 for the top 10 most productive authors). Notably, their high productivity can be explained by a large number of invited publications: 17 of Lingard’s articles were from the Writer’s Craft series and six of Leppink’s articles were from the Statistical Points and Pitfalls series. All of these articles were invited by the Editor-in-Chief, led by Lingard or Leppink, and peer reviewed.

**Table 3:** Top 10 most productive authors (*WoS*) and their collaboration data

Author (predicted gender)	Cluster affiliation	Number of articles	Number of co-authors	Collaboration strength	Average citations
Lingard, L (W)	10	20	25	31	3.8
Leppink, J (M)	9	16	8	23	11.2
Ten Cate, O (M)	7	13	40	44	16.6
Varpio, L (W)	1	12	30	37	4.8
Van der Vleuten, C (M)	3	11	21	27	16.5
Watling, C (M)	6	11	15	16	13.4
Durning, S (M)	3	10	32	44	4.8
O’Sullivan, P (W)	9	10	6	20	8.1
Winston, K (M)	9	10	18	33	6.4
Cleland, J (W)	10	9	29	32	4.2

Collaboration strength represents the number of unique co-authors with whom a given author has published in PME over the period analyzed

*W* woman, *M* man

PME received manuscripts from authors in 81 countries. US-based authors submitted the most (*n* = 572; 24.7%), followed by the UK (*n* = 312; 15.0%) and the Netherlands (*n* = 276; 13.3%). Notably, in 2019, the journal received its first submissions from authors based in Cuba, Cyprus, Lebanon, Oman, and Zimbabwe. PME articles listed affiliations in 35 countries with the largest number of authors based in the Netherlands (*n* = 151; 29.3%), US (*n* = 111; 21.5%) and Canada (*n* = 110; 21.3%).

In the next sections, to visualize author collaborations, we present network maps illustrating relationships based on different levels of co-authorship.

**Fig. 2:**
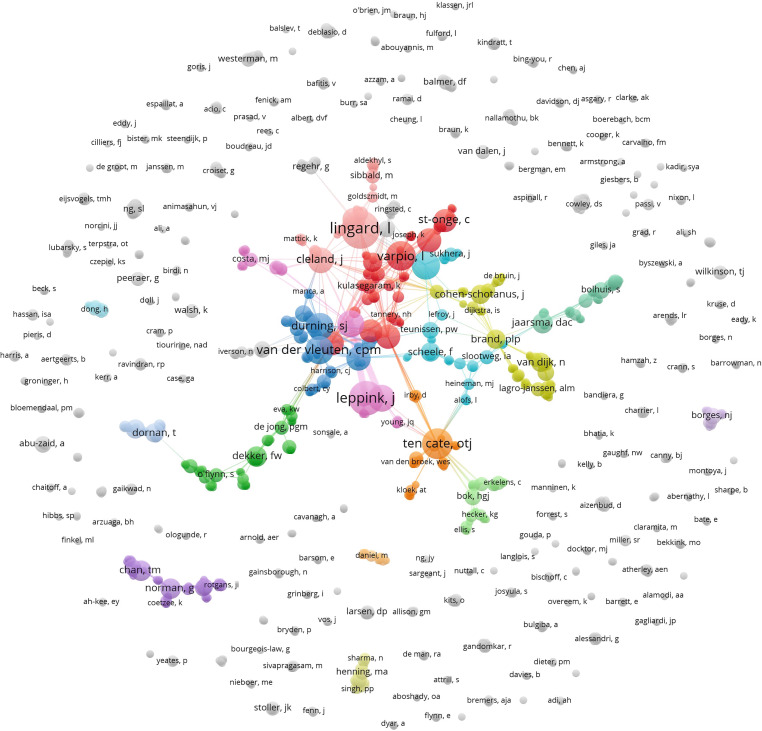
Co-authorship network of 1227 authors who published in PME from 2012–2019. Node size indicates number of documents, node color represents cluster affiliation of author. Clusters with less than 15 authors are in *grey*

PME authors were quite collaborative. The authors with the largest number of co-authors were Ten Cate, Durning, and Maggio, who collaborated with 40, 33 and 33 different authors, respectively. Based on the co-authorship network displayed in the center of Fig. 2, 447 of the 1227 authors were part of the largest component in the co-authorship network. This means that these 447 authors were connected either directly (through co-authoring at least one article together) or indirectly (by having a co-author in common). On the other hand, there were 57 clusters, positioned at the periphery of the network, composed of individual authors who published all their work in PME as single authors.

Authors were grouped into 220 clusters (Fig. 2). The largest cluster (red, right of center of the network) contains 57 authors with Varpio (12 publications), Driessen (8), Maggio (8), Young (8), Artino (7), St Onge (7), and Paradis (5) being the most productive. This cluster contains 56% women and 38% men (6% of authors’ gender could not be determined). The second largest cluster (dark green, top left of center) consists of 52 authors who, on average, published less frequently in PME: Dekker published 6 articles, followed by O’Flynn, Cantillon-Murphy, De Jong, Ommering, and Dankbaar with 3 PME publications each. This cluster is slightly more male dominated with 53% men and 45% women authors (2% of authors’ gender could not be determined). The third cluster (dark blue, bottom left of center) connects 47 authors, the most prominent being Van der Vleuten, Durning, and Schuwirth with 11, 10 and 8 articles, respectively. This cluster contained 49% authors with male first names and 45% with female first names (6% of authors’ gender could not be determined).

When considering these three dominant co-author clusters, it is interesting to note that many of these authors are leaders of, and faculty members in, units that administer graduate programs in HPE. For example, at the time of this data extraction, Durning, Maggio, Varpio, and Artino were faculty members in the Uniformed Services University’s HPE graduate program. Such graduate programs in HPE, many of which include publication requirements in order to graduate and would likely include faculty member co-authors, have the potential to increase an author’s opportunities for collaborative authorship. Additionally, we see in many of these clusters Dutch members who were key in PME’s founding and who have played leadership roles in the NVMO.

#### For discussion

What are additional interpretations of the co-authorship relationships in Fig. 2?

### Affiliations

**Fig. 3:**
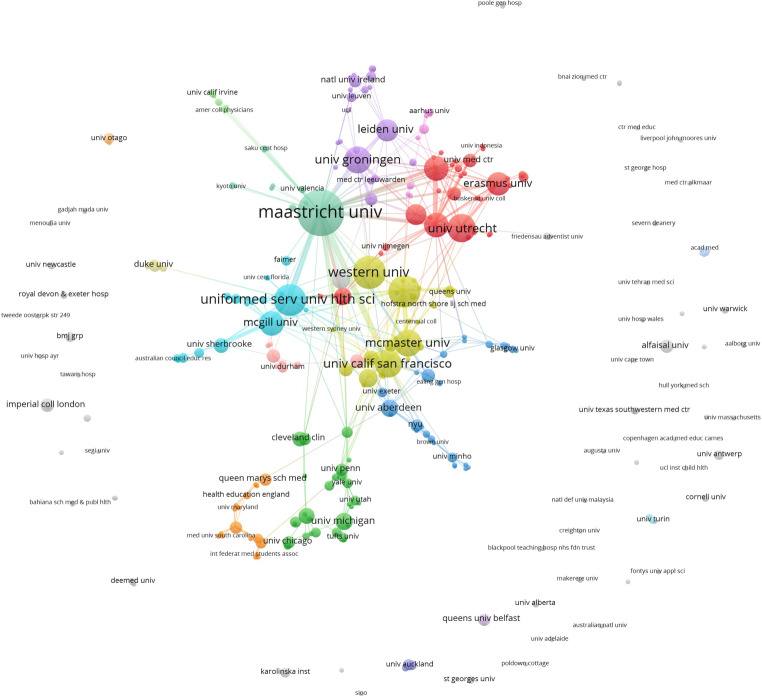
Collaboration network of 333 institutions whose authors published in PME from 2012–2019. Node size indicates number of documents, node color represents cluster affiliation of institution. Clusters with less than 4 institutions are in *grey*

Authors were affiliated with 333 different institutions, 233 of which were connected in the largest component of the institutional collaboration network. Authors affiliated with Maastricht University published the most articles (*n* = 65), followed by Western University (*n* = 34) and University of Toronto (*n* = 32) (see Tab. 3 for top 10 institutions). Institutional clusters often reflect close domestic collaboration. For example, in Fig. 3, the largest cluster (red; to the right of center) includes 36 institutions, with three Dutch universities—University of Utrecht (*n* = 25), Radboud University Nijmegen (*n* = 19) and Vrije University (*n* = 19)—being the most prolific. The second largest cluster (green; bottom center) includes 30 institutions, the most prolific of which are in the US, including University of Michigan (*n* = 9), Harvard University (*n* = 8), and Cleveland Clinic (*n* = 7). The third largest cluster contained 28 institutions, led by University of Aberdeen (*n* = 9), University of Liverpool (*n* = 6), and New York University (*n* = 5).

PME articles included authors with institutional affiliations in 40 countries. The great majority of PME articles were written by authors based in the Netherlands (171 publications), reflecting the former national focus of the journal. The US (138), Canada (131), and UK (76) are the second, third and fourth most represented countries, respectively. In terms of collaboration patterns, these countries showed the strongest ties with each other. The largest number of co-publications occurred between the Netherlands and the US (24), the US and Canada (16), and Canada and the UK (10). We identified that authors from nine countries represented only collaborated with authors from their own country. See Zenodo file for the top 10 institutions by number of articles published [23].

The presence of these predominantly Western institutional and geographical affiliations aligns with patterns observed in HPE more broadly [26–28], although authors from the Netherlands are more heavily represented. The prevalence of Dutch authors and editorial board members is likely an artifact of the journal’s history; that is, the journal originated in the Netherlands, where it remains today, and is sponsored by the NVMO.

#### For discussion

What other factors could account for the institutional and international collaboration depicted in Fig. 3?

### Topics

Author keywords were available for 401 of the 518 articles. After cleaning terms (see Methods), 955 unique terms were used to describe these documents. The most frequent terms were medical education (assigned to 73 documents), medical students (26), assessments (24), feedback (21), undergraduate medical education (19) and curriculum (15). As one might expect, the distribution of documents per author keyword was very skewed, and the majority of terms (746, 78%) were assigned to one document only.

**Fig. 4:**
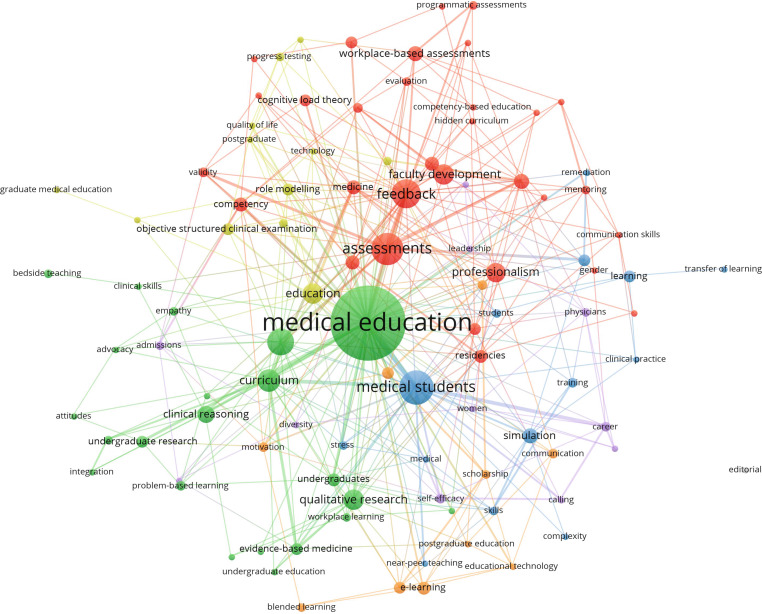
Co-occurrence network of 101 most frequent author keywords. Node size indicates number of documents (at least 3), node color represents cluster affiliation of keyword

Fig. 4, which presents a network of keywords that co-occur, is limited to author keywords assigned to a minimum of 3 documents and thus reduced from 955 to the 101 most frequent keywords. As the most frequent keyword that co-occurred with the largest number of other keywords, *medical education*, is at the center of the network as the largest node. It is part of the second largest cluster 2 (green) together with 21 other author keywords, including undergraduate medical education (19 documents), curriculum (15), qualitative research (13) and clinical reasoning (10). Overall, the network contained 7 clusters. The largest cluster 1 (red) contains 31 different keywords with assessments (24), feedback (21), faculty development (13) and professionalism (12) being the most frequent. The third cluster (blue) contained 14 keywords, including medical students (26), simulation (9), competency-based medical education (6) and learning (6).

In considering these clusters, it is easy to be distracted by the central key word *medical education*. The dominance of this keyword possibly indicates that PME is more focused on manuscripts addressing topics specific to training of doctors and less so on the broader field of HPE. However, in our experience, the term medical education is often used interchangeably with the more inclusive term, HPE, and so this finding should be interpreted with caution. Next, it is noticeable that the largest cluster (red) contains keywords that one could group as associated with assessment (e.g., assessments, workplace-based assessments, programmatic assessment), suggesting that PME has made assessment a key focus. While these keyword clusters can be interesting, these data speak to the lack of consistency in author keyword selection, which makes this type of network map less useful. This lack of consistency reflects similar findings in a case study of the multiple terms HPE researchers use for the term faculty [29].

#### For discussion

In what ways, if any, do you find these clusters surprising?

Are there any topics that you think are missing?

#### Discussion: future steps

Upon reflection of our findings from this case study, below we address four key takeaways for PME: growing thoughtfully, diversifying, educating, and liberating and sharing journal data.

### Growing thoughtfully

While the number of manuscripts that PME published has remained constant, there has been a significant increase in the number of submissions, which have continued to climb during and beyond the study period. In 2021, for example, PME received over 900 submissions, which may be an artifact of the COVID-19 pandemic. But in 2022 PME submissions are on pace to exceed this number. As noted, journal pages are precious, and each article exacts a cost that demands both monetary resources (e.g., the publisher’s estimated cost to produce an article is between $ 3500–4000 [30]) and human resources (e.g., peer review and editorial oversight). At PME, we would like to grow the number of published articles to enable a broader diversity of perspectives and to raise the journal’s acceptance rate. Over the past year, we have started to address this desire by encouraging authors to streamline their manuscripts (i.e., shorter word counts and fewer figures and tables). Where appropriate, we also have suggested that authors consider depositing supplemental materials or extended methods sections on repository sites like Zenodo. Such practices provide authors with additional space to fully describe their approaches and share multiple exhibits, while also producing a digital object identifier (DOI), a persistent identifier that is searchable and citable. Such practices also support an open-science approach to medical education research, which we believe encourages the responsible conduct of research [6]. Furthermore, looking to the future, we are investigating the possibility of alternate journal models that would enable us to expand our allowable number of published pages.

### Diversifying

While PME received and published manuscripts from around the world, the majority of those manuscripts, as well as the majority of the researchers who peer reviewed those manuscripts, were affiliated with Western countries. While this finding aligns with the broader literature [26, 28] and statistics from other journals in the field (e.g., *Medical Education* [31]), we feel there is an urgent need to grow our global representation and, as Kusurkar recently wrote in a PME editorial, work toward fixing the “leaky pipeline” of medical education researchers [32]. To this end, we are strategizing with our editorial team and editors of other HPE journals on how to more fully involve authors, readers, reviewers, and editors from around the globe.

### Educating

Educating the medical education community of scholars has always been one of PME’s most cherished goals, and based on several of the highly cited, education-focused articles listed in Tab. 2, it seems our education goal is also valued by the community. For example, the Writer’s Craft, Insiders’ Perspective, and Statistical Pitfalls, are all publication types that take an educational focus, and these articles have been some of our most popular collections, as measured by downloads and even social media attention. We plan to continue to create novel article types that not only expand authors’ publication options but also serve as open-access educational materials that can be broadly applied across the community to improve the field’s research and communication. Additionally, we have been increasingly offering topical and skills-based workshops for the HPE community that have covered areas such as responding to peer reviewers, responsibly conducting research, and targeting the right journal for your manuscript. We will continue to seek opportunities to educate members of our HPE community. Additionally, in an attempt to offer more formal educational opportunities, we are in the early phases of creating an editorial internship program that will familiarize researchers with the editorial process and the responsibilities of being an editor.

### Liberating and sharing journal data

In this study, we were fortunate to have access to the Editorial Manager’s internal report system; we also had permission from Springer to use those data. Notwithstanding this access and permission, we currently do not have standing, guaranteed, real-time access to our own data—data that are generated by our readers and editors. This lack of access is a policy hurdle, not a technical hurdle. For example, the Open Journal System, which is operated by the Public Knowledge Project, is the world’s largest open access publishing platform. It makes readily available—in real time—data on article and abstract views, editorial and review activity, and user counts by submission. And these data are readily available to its editors without the need for a special request [33]. We contend that access to a journal’s data is critical to monitoring the journal’s health and assessing the success of new initiatives. For example, if a journal implements a program to train researchers from under-represented countries, we would want to know if and how successful that initiative is at improving the representation of those countries in the scholarly conversation. Moreover, we advocate for the widespread capability to share such data so that all members of the community can learn from these efforts. If the editors have access to these data and can share the data with their constituents, then together we can work to identify what works and what does not across medical education’s publishing landscape.

## Conclusion

In this case study, we aimed to provide a glimpse into the inner workings of PME for a variety of stakeholders and to generate evidence to guide PME’s next steps. As a way of “taking stock” of where we are and where we have been, we hope this analysis can help guide PME’s future. In considering this future, it seems that some of the following topics may be worthy of broader consideration by the HPE community: growing PME thoughtfully; diversifying and educating editorial teams, authors, and reviewers; and liberating and sharing journal data. We are optimistic that this case study will kick start the conversation on these topics for PME and other journals in the field.

## References

[1] Ioannidis JPI (2005). Why most published research findings are false. PLoS Med.

[2] Ioannidis JPI (2018). Meta-research: why research on research matters. PLoS Biol.

[3] Peterson D, Panofsky A. Metascience as a scientific social movement. Version 1. SocArXiv. 2020. https://osf.io/preprints/socarxiv/4dsqa/. Accessed 3 Feb 2022.

[4] Fanelli D (2009). How many scientists fabricate and falsify research? A systematic review and meta-analysis of survey data. Plos One.

[5] Marusic A, Wager E, Utrobicic A, Rothstein HR, Sambunjak D (2016). Interventions to prevent misconduct and promote integrity in research and publication. Cochrane Database Syst Rev.

[6] Artino AR, Driessen EW, Maggio LA (2019). Ethical shades of gray: international frequency of scientific misconduct and questionable research practices in health professions education. Acad Med.

[7] Lee CJ, Sugimoto CR, Zhang G, Cronin B (2013). Bias in peer review. J Am Soc Inf Sci Technol.

[8] Maggio LA, Bynum WE, Schreiber-Gregory DN, Durning SJ, Artino AR (2020). When will I get my paper back? A replication study of publication timelines for health professions education research. Perspect Med Educ.

[9] Himmelstein D. The history of publishing delays. Satoshi Village: the blog of Daniel Himmelstein. 2016. https://blog.dhimmel.com/history-of-delays/. Accessed 3 Feb 2022.

[10] Piwowar H, Priem J, Larivière V (2018). The state of OA: a large-scale analysis of the prevalence and impact of Open Access articles. Peer J.

[11] BMJ. Evidence based publishing.. https://www.bmj.com/about-bmj/evidence-based-publishing. Accessed 3 Feb 2022.

[12] PLOS.. https://plos.org/about/. Accessed 3 Feb 2022.

[13] Otlet P (1934). Traité de documentation. Le Livre sur le Livre: Théorie et Pratique.

[14] Garfield E (1955). Citation indexes for science; a new dimension in documentation through association of ideas. Science.

[15] Sugimoto CR, Larivière V (2018). Measuring research: what everyone needs to know.

[16] Demografix ApS.. https://genderize.io/. Accessed 3 Feb 2022.

[17] Hornstein P, Tuyishime H, Mutebi M, Lasebikan N, Rubagumya F, Fadelu T (2022). Authorship equity and gender representation in global oncology publications. JCO Glob Oncol.

[18] Bhatia S, Cotton CC, Kim E (2022). Gender and nationality trends in manuscripts published in prominent gastroenterology journals between 1997 and 2017. Dig Dis Sci.

[19] Maggio LA, Ninkov A, Costello JA, Driessen EW, Artino AR (2021). Knowledge syntheses in medical education: meta-research examining author gender, geographic location, and institutional affiliation. PLoS ONE.

[20] Google (2022). Google sheets.

[21] Microsoft Corporation. Microsoft Excel. Version 16.59. 2022. https://office.microsoft.com/excel. Accessed 1 June 2022.

[22] van Eck NJ, Waltman L. VOSViewer: Visualizing Scientific Landscapes. 2010. https://www.vosviewer.com. Accessed 1 June 2022.

[23] Maggio LA, Haustein S, Costello JA, Driessen EW, Artino AR (2022). Perspectives on medical education. J Data Suppl Files.

[24] Varpio L, Nagler A (2017). Perspectives on Medical Education Special Edition : Lessons learned from health professions education scholarship failures surprises. Perspect Med Educ.

[25] Maggio LA, Costello JA, Norton C, Driessen EW, Artino AR (2021). Knowledge syntheses in medical education: a bibliometric analysis. Perspect Med Educ.

[26] Maggio LA, Costello JA, Ninkov A, Frank JR, Artino AR (2022). The voices of medical education science: describing the published landscape.

[27] Madden C, O’Malley R, O’Connor P, O’Dowd E, Byrne D, Lydon S (2021). Gender in authorship and editorship in medical education journals: a bibliometric review. Med Educ.

[28] Thomas EG, Jayabalasingham B, Collins T, Geertzen J, Bui C, Dominici F (2019). Gender disparities in invited commentary authorship in 2459 medical journals. JAMA Netw Open.

[29] Teunissen PW, Atherley A, Cleland JJ (2022). Advancing the science of health professions education through a shared understanding of terminology: a content analysis of terms for “faculty”. Perspect Med Educ.

[30] Van Noorden R (2013). Open access: The true cost of science publishing [published correction appears in Nature. 2013;496:151] [published correction appears in Nature. 2013;499:19. Nature.

[31] Ajjawi R, Crampton PES, Ginsburg S (2022). Promoting inclusivity in health professions education publishing. Med Educ.

[32] Kusurkar RA (2022). The leaky pipeline of publications and knowledge generation in medical education. Perspect Med Educ.

[33] Public Knowledge Project. Statistics.. https://docs.pkp.sfu.ca/admin-guide/en/statistics. Accessed 27 Mar 2022.

[34] Neubauer (2019). Neubauer, et al. How phenomenology can help us learn from the experiences of others. Perspect Med Educ.

[35] Varpio (2018). Varpio. Using rhetorical appeals to credibility, logic, and emotions to increase your persuasiveness. Perspect Med Educ.

[36] Artino (2012). Artino. Academic self-efficacy: from educational theory to instructional practice. Perspect Med Educ.

[37] Lefroy (2015). Lefroy, et al. Guidelines: the do’s, don’ts and don’t knows of feedback for clinical education. Perspect Med Educ.

[38] Peters (2014). Peters, et al. Bedside teaching in medical education: a literature review. Perspect Med Educ.

[39] Kamphuis (2014). Kamphuis. Augmented reality in medical education?. Perspect Med Educ.

[40] Kogan (2017). Kogan. Guidelines: The do’s, don’ts and don’t knows of direct observation of clinical skills in medical education. Perspect Med Educ.

[41] Leppink (2015). Leppink. The evolution of cognitive load theory and its application to medical education. Perspect Med Educ.

